# Development and Validation of a Machine Learning Algorithm for Predicting Diabetes Retinopathy in Patients With Type 2 Diabetes: Algorithm Development Study

**DOI:** 10.2196/58107

**Published:** 2025-02-07

**Authors:** Sunyoung Kim, Jaeyu Park, Yejun Son, Hojae Lee, Selin Woo, Myeongcheol Lee, Hayeon Lee, Hyunji Sang, Dong Keon Yon, Sang Youl Rhee

**Affiliations:** 1 Department of Family Medicine, Kyung Hee University Medical Center, Kyung Hee University College of Medicine, Seoul, Republic of Korea; 2 Center for Digital Health, Medical Science Research Institute, Kyung Hee University College of Medicine, 23 Kyungheedae–ro, Dongdaemun–gu, Seoul, 02447, Republic of Korea, 82 1091565964, 82 29610680; 3 Department of Regulatory Science, Kyung Hee University, Seoul, Republic of Korea; 4 Department of Precision Medicine, Kyung Hee University College of Medicine, Seoul, Republic of Korea; 5 Department of Endocrinology, Kyung Hee University Medical Center, Kyung Hee University College of Medicine, Seoul, Republic of Korea; 6 Department of Pediatrics, Kyung Hee University Medical Center, Kyung Hee University College of Medicine, Seoul, Republic of Korea

**Keywords:** type 2 diabetes, diabetes retinopathy, algorithm, machine learning, prediction, comorbidities, retinal, ophthalmology

## Abstract

**Background:**

Diabetic retinopathy (DR) is the leading cause of preventable blindness worldwide. Machine learning (ML) systems can enhance DR in community-based screening. However, predictive power models for usability and performance are still being determined.

**Objective:**

This study used data from 3 university hospitals in South Korea to conduct a simple and accurate assessment of ML-based risk prediction for the development of DR that can be universally applied to adults with type 2 diabetes mellitus (T2DM).

**Methods:**

DR was predicted using data from 2 independent electronic medical records: a discovery cohort (one hospital, n=14,694) and a validation cohort (2 hospitals, n=1856). The primary outcome was the presence of DR at 3 years. Different ML-based models were selected through hyperparameter tuning in the discovery cohort, and the area under the receiver operating characteristic (ROC) curve was analyzed in both cohorts.

**Results:**

Among 14,694 patients screened for inclusion, 348 (2.37%) were diagnosed with DR. For DR, the extreme gradient boosting (XGBoost) system had an accuracy of 75.13% (95% CI 74.10‐76.17), a sensitivity of 71.00% (95% CI 66.83‐75.17), and a specificity of 75.23% (95% CI 74.16‐76.31) in the original dataset. Among the validation datasets, XGBoost had an accuracy of 65.14%, a sensitivity of 64.96%, and a specificity of 65.15%. The most common feature in the XGBoost model is dyslipidemia, followed by cancer, hypertension, chronic kidney disease, neuropathy, and cardiovascular disease.

**Conclusions:**

This approach shows the potential to enhance patient outcomes by enabling timely interventions in patients with T2DM, improving our understanding of contributing factors, and reducing DR-related complications. The proposed prediction model is expected to be both competitive and cost-effective, particularly for primary care settings in South Korea.

## Introduction

The global prevalence of diabetes mellitus (DM) in 2019 was estimated to be 463 million and is expected to reach 700 million by 2045 [1]. Diabetic retinopathy (DR) is the most common complication of DM and a leading cause of preventable blindness in adults [2-4]. The longer the period of diabetes and the lower the blood sugar control, the higher the risk of DR. Particularly, in patients with diabetes for >10 and >15 years, the prevalence of DR was 46.2% and 66.7%, respectively [5]. Additionally, the risk of DR increases by 1.4-fold for each 1% increase in glycated hemoglobin (HbA_1c_) levels. Moreover, alongside well-established risk factors, such as poor glycemic control and prolonged diabetes duration, it is important to acknowledge that DR can also manifest in patients with a relatively lower BMI.

DR progression risk factors include both correctable and noncorrectable factors [6,7]. Although DR can be continuously examined and prevented from the beginning, by the time a patient with diabetes experiences symptoms, such as decreased vision due to DR, the disease has already progressed significantly, and serious complications have occurred. Accordingly, the US Centers for Disease Control and Prevention recommends that patients undergo regular tests for DR, even those without symptoms, and receive appropriate management for any identified abnormalities [8,9]. Therefore, patients with diabetes are widely recommended to undergo routine testing annually to prevent DR progression [9].

DR testing requires significant time and effort. However, shortages in trained professionals have been reported. According to a 2022 survey, only 46% of patients with DM in South Korea underwent fundus examination, with examination rates in the mid to high 30% for patients in their 30s to 50s [10]. Effective DR management requires individualized risk models and tools to predict and identify the cause of disease onset. Developing these models can allow for a better prediction of DR risk and improved screening efficiency. DR progression can be prevented using a DR management strategy that focuses on high-risk individuals.

## Methods

### Study Population and Data Collection

**Figure 1.**
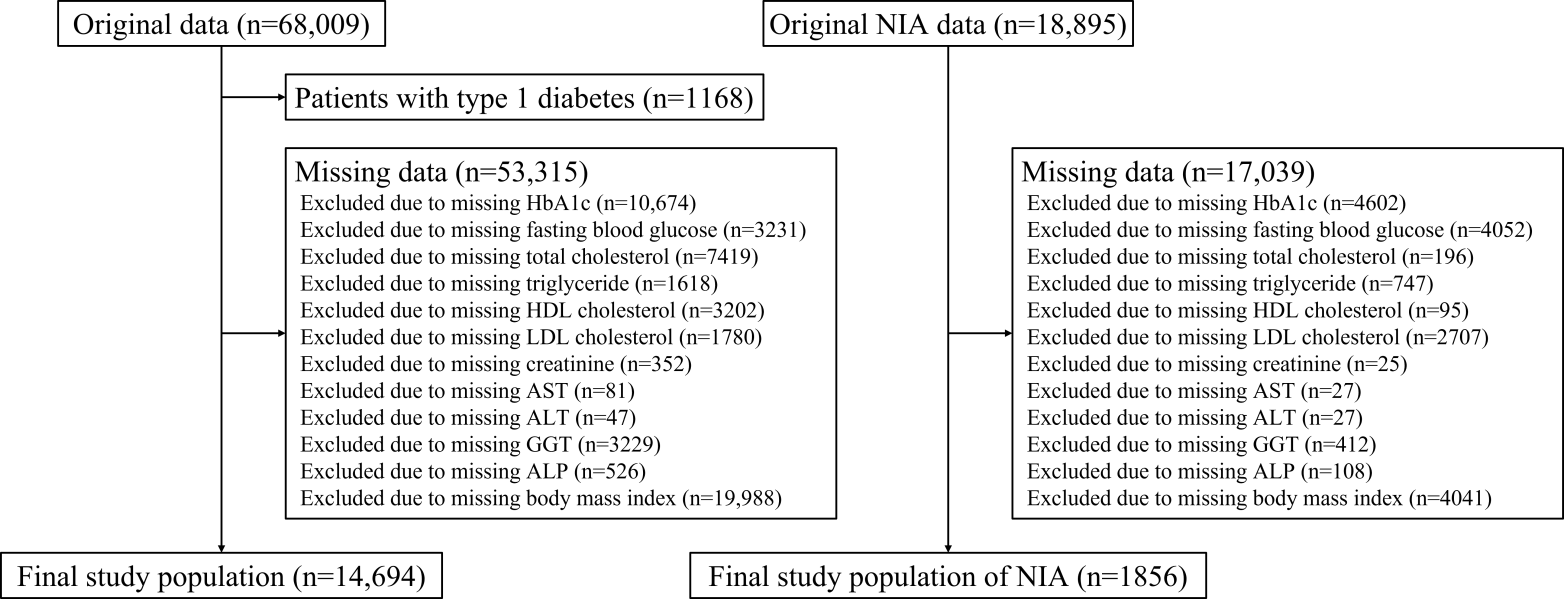
Study workflow. ALP: alkaline phosphatase; ALT: alanine transaminase; AST: aspartate transaminase; GGT: γ-glutamyl transferase; HbA_1c_: glycated hemoglobin; HDL: high-density lipoprotein; LDL: low-density lipoprotein; NIA: National Information Society Agency.

This retrospective study used data from 2 independent longitudinal cohorts as part of an observational study. Data were collected from a hospital between January 1, 2008, and December 31, 2022. Appropriate participants were selected from patients with type 2 diabetes mellitus (T2DM), excluding those with type 1DM (T1DM) and prior cases of DR. Finally, 14,694 patients from a tertiary hospital at Kyung Hee University Medical Center were included in the discovery cohort. Extravalidation data were acquired from a retrospective dataset from Kyung Hee University Hospital at Gangdong and Gachon University Gil Hospital, which included 1856 suitable patients (Figure 1).

### Input Variables

The model encompasses an exhaustive set of 57 variables, including demographic variables such as age, sex, and BMI [11]. Medical history included comorbidities (hypertension and dyslipidemia), macrovascular complications (cardiovascular disease [CVD], dementia, Parkinson disease, and microangiopathic disease), microvascular complications (chronic kidney disease [CKD] and neuropathy), end-stage renal disease, and cancer.

The medication history variables considered the types of drugs used to treat DM (metformin, sulfonylurea, dipeptidyl peptidase-4 inhibitors, meglitinide, thiazolidinedione, α-glucosidase inhibitors, insulin, glucagon-like peptide-1 receptor agonists, and sodium-glucose cotransporter 2 inhibitors), hypertension (angiotensin II receptor blockers, angiotensin-converting enzyme inhibitors, calcium channel blockers, diuretics, and β-blockers), and dyslipidemia (statin, fibrate, and ezetimibe), as well as antiplatelet drugs (aspirin, clopidogrel, cilostazol, and glycoprotein IIb/IIIa antagonists). Blood tests were performed to measure HbA_1c_, fasting blood glucose, total cholesterol, triglyceride, high-density lipoprotein cholesterol, low-density lipoprotein cholesterol, serum creatinine, aspartate aminotransferase, alanine aminotransferase, γ-glutamyl transferase, and alkaline phosphatase. The median and SD of each parameter were used as input variables for the BMI and blood test results. Demographic variables such as age, sex, and BMI were collected at the patient’s initial visit to the clinic. Blood tests (HbA_1c_, fasting blood glucose, and total cholesterol) were conducted during regular follow-up visits, with the last recorded value before the onset of DR used for analysis.

### Identification of New DR Cases

New-onset DR in patients with T2DM was identified using the *ICD-10* (*International Statistical Classification of Diseases, Tenth Revision*) codes for retinopathy (E11.3-E14.3). T2DM was diagnosed as an anchoring event, and subsequent data collection, including DR identification, was based on the patient’s first recorded diagnosis of diabetes. As relying solely on electronic health record data to identify conditions has inherent limitations in terms of accuracy, *ICD-10* codes were used to confirm whether the patients were diagnosed with retinopathy. This approach allowed for more reliable identification of retinopathy cases in this cohort.

### Data Preprocessing

The data were preprocessed in 3 steps. First, individuals with T1DM were excluded to focus on those with T2DM using electronic health record data and identifying patients with T1DM via the *ICD-10* code (E10). Second, patients with a prior diagnosis of DR were excluded and identified using the *ICD-10* codes for retinopathy (E11.3-E14.3). Thirdly, patients with missing data in categories such as demographics, physical examination results, blood test results, medication history, or comorbidities were excluded from the analysis. Data from physical examinations and blood tests conducted before the onset of DR were analyzed. The most recent values recorded before DR onset were used and averaged across the study period for each patient. Covariates, including medication history and comorbidities, were collected at the patient’s first visit and incorporated into the analysis. The final dataset represents variables as means (SD) or numbers (percentages), as appropriate.

### Model Training and Validation

The conventional machine learning (ML) methodology for prediction tasks requires splitting the data into training and testing sets. However, the available data on the 3-year incidence of DR are insufficient in the context of this study. Therefore, the model was trained on the complete dataset rather than dividing it for internal validation. A separate external dataset, the National Information Society Agency, was used to assess the model’s generalizability. This method was vital for confirming the robustness of the model for new unencountered data.

### Model Development

This study applied decision-tree-based ensemble models, including AdaBoost, CatBoost, light gradient-boosting machine (LightGBM), random forest, and extreme gradient boosting (XGBoost). To optimize each model’s performance, hyperparameter tuning was conducted using GridSearchCV to achieve the highest area under the receiver operating characteristic (ROC) curve (AUROC) and determine the most practical combination of hyperparameters.

### ML Analysis

Tree-based and linear classification models were used to establish AUROC scores to predict the occurrence of DR. After determining the optimal hyperparameters, the model was trained to ensure accurate prediction. Given the class imbalance in the data, a synthetic minority oversampling technique was used to generate synthetic samples. The model’s performance was evaluated using various metrics, such as AUROC, accuracy, sensitivity, specificity, and balanced accuracy, which were calculated based on the probability predictions generated by the model. A 10-fold stratified cross-validation was used to assess the model’s ability to process new data, and the Youden Index was used within each stratification to pinpoint the optimal threshold [12]. The mean and 95% CIs for each performance metric were calculated to determine the average performance and variability of the model. The model performance was graphically represented by an ROC curve supplemented by the mean ROC curve and SD within that range, illustrating the model performance distribution. The XGBoost model, which provided the highest AUROC score among the various decision-tree models examined, was selected to determine the most crucial features for predicting DR. The significance of each feature was extracted by leveraging the important attributes of the XGBoost model. The top 15 features with the most significant impacts on the model were identified and plotted to visualize their effects on the predictions.

### Performance Metrics

Five metrics were used to evaluate the performance of the proposed model: AUROC, accuracy, sensitivity, specificity, and balance accuracy. The AUROC is a resilient performance measure that assesses a model’s ability to distinguish between classes across all potential thresholds. Resilience stems from the consideration of both sensitivity and specificity, making it the preferred metric, particularly in scenarios where classes are imbalanced. Accuracy is a simple and intuitive performance metric that denotes the ratio of accurate results (both true positives and negatives) to the total number of cases examined. However, when used alone, the accuracy can be misleading, particularly for unbalanced datasets, thereby necessitating additional performance metrics. The sensitivity and specificity were used to evaluate the ability of the model to correctly identify positive and negative cases, respectively. Sensitivity measures the ability of a model to identify positive cases correctly. By contrast, specificity measures the ability of the model to correctly identify negative cases, indicating its ability to avoid false-positive findings. Finally, balanced accuracy was included to provide a more balanced view of the model performance, particularly when dealing with class imbalances. Balanced accuracy is an excellent alternative to accuracy for unbalanced datasets because it assigns equal weights to sensitivity and specificity. Combining these metrics enabled an assessment of the performance of the model from different perspectives, ensuring a more comprehensive evaluation [13,14].

### Software and Libraries

All data preprocessing, model creation, and analyses were performed using Python (version 3.9.16; Python Software Foundation). The libraries used to execute the ML algorithms and manipulate the data included Scikit-learn 1.2.2, NumPy 1.23.5, and Pandas 1.5.3. For data visualization, Matplotlib 3.7.1 and Seaborn 0.12.2 were used.

### Ethical Considerations

This study was approved as exempt by the institutional review board of Kyung Hee University Hospital because of the use of deidentified patient data in a secure environment (approval number KHSIRB-22-473(EA)).

## Results

### Cohort Baseline Characteristics

Among the 14,694 patients in the discovery cohort, the mean age was 62.8 (11.24) years with 7730 (52.61%) males, and the mean BMI was 25.1 (3.58) kg/m^2^. The discovery cohort identified 348 (2.37%) patients as having DR. For additional validation, 1856 patients were included, of which 1053 (56.73%) patients were male, and the mean age was 57.7 (11.88) years. A total of 137 patients (7.38%) were identified as having DR within this validation cohort (Table 1 and Figure 1).

**Table 1.** Baseline characteristics of the study and extra-validation datasets.

Study dataset							Extra-validation dataset			
	Total (N=14,694)		Control (n=14,346)			Case^a^ (n=348)		Total (n=1856)		Control (n=1719)	Case (n=137)
Age (years), mean (SD)	62.8 (11.24)		62.9 (11.26)			60.2 (10.15)		57.7 (11.74)		57.6 (11.88)	58.4 (9.92)
Male, n (%)	7730 (52.61)		7555 (52.66)			175 (50.29)		1053 (56.73)		976 (56.78)	77 (56.20)
Female, n (%)	6964 (47.39)		6791 (47.34)			173 (49.71)		803 (43.27)		743 (43.22)	60 (43.80)
BMI (kg/m^2^), mean (SD)	25.1 (3.58)		25.1 (3.58)			24.6 (3.29)		25.2 (3.45)		25.2 (3.49)	24.6 (2.84)
Blood test, mean (SD)											
HbA_1c_^b^ (%)	6.9 (0.90)		6.9 (0.89)			7.3 (0.91)		7.2 (1.07)		7.2 (1.08)	7.3 (0.94)
Fasting blood glucose (mg/dL)	146.6 (38.12)		146.4 (38.08)			154.8 (39.09)		141.8 (38.06)		142.1 (38.05)	138.1 (38.12)
Total cholesterol (mg/dL)	155.7 (30.16)		155.8 (30.17)			150.3 (29.30)		158.1 (29.82)		158.2 (29.96)	156.9 (28.04)
Triglyceride (mg/dL)	140.8 (63.09)		141.1 (63.28)			130.9 (53.97)		145.0 (63.33)		145.4 (63.90)	141.1 (55.78)
HDL^c^ cholesterol (mg/dL)	47.5 (11.34)		47.5 (11.35)			47.6 (10.80)		44.7 (9.78)		44.7 (9.76)	44.9 (10.03)
LDL^d^ cholesterol (mg/dL)	88.1 (24.22)		88.2 (24.24)			84.1 (22.94)		88.2 (27.70)		88.6 (27.82)	83.9 (25.77)
Creatinine (mg/dL)	0.9 (0.45)		0.9 (0.45)			1.0 (0.59)		1.1 (1.00)		1.1 (1.02)	1.1 (0.72)
AST^e^ (U/L)	26.3 (11.19)		26.3 (11.25)			23.8 (8.19)		24.3 (8.12)		24.4 (8.16)	23.2 (7.50)
ALT^f^ (U/L)	23.7 (12.13)		23.8 (12.19)			20.7 (9.11)		24.9 (12.55)		25.1 (12.77)	21.9 (8.81)
GGT^g^ (U/L)	37.6 (33.86)		37.7 (33.97)			32.6 (28.37)		37.9 (32.24)		38.4 (32.68)	31.8 (25.39)
ALP^h^ (U/L)	77.9 (23.03)		77.9 (23.05)			77.7 (22.02)		148.5 (88.93)		147.2 (89.21)	164.4 (83.97)
Comorbid conditions, n (%)											
Hypertension	8522 (58.00)		8367 (58.32)			155 (44.54)		1209 (65.14)		1138 (66.20)	71 (51.82)
Dyslipidemia	9343 (63.58)		9217 (64.25)			126 (36.21)		1004 (54.09)		946 (55.03)	58 (42.34)
Macrovascular complications, n (%)											
Cardiovascular disease^i^	7343 (49.97)		7216 (50.30)			127 (36.49)		785 (42.30)		747 (43.46)	38 (27.74)
Dementia	14,515 (98.78)		178 (1.24)			1 (0.29)		83 (4.47)		82 (4.77)	1 (0.73)
Parkinson disease	279 (1.90)		279 (1.94)			N/A		13 (0.70)		13 (0.76)	N/A^j^
Microangiopathic disease^k^	102 (0.69)		99 (0.69)			3 (0.86)		336 (18.10)		321 (18.67)	15 (10.95)
Microvascular complications, n (%)											
ESRD^l^	135 (0.92)		132 (0.92)			3 (0.86)		155 (8.35)		151 (8.78)	4 (2.92)
Neuropathy	4657 (31.69)		4564 (31.81)			93 (26.72)		543 (29.26)		508 (29.55)	35 (25.55)
Cancer	2755 (18.75)		2734 (19.06)			21 (6.03)		249 (13.42)		246 (14.31)	3 (2.19)
Medication use, n (%)											
Diabetes mellitus^m^											
Metformin	8374 (56.99)		8147 (56.79)			227 (65.23)		558 (30.06)		503 (29.26)	55 (40.15)
Sulfonylurea	5186 (35.29)		5029 (35.06)			157 (45.11)		218 (11.75)		191 (11.11)	27 (19.71)
DPP-4^n^ inhibitor	3226 (21.95)		3150 (21.96)			76 (21.84)		94 (5.06)		88 (5.12)	6 (4.38)
Meglitinide	802 (5.46)		763 (5.32)			39 (11.21)		78 (4.20)		65 (3.78)	13 (9.49)
Thiazolidinedione	1062 (7.23)		1012 (7.05)			50 (14.37)		17 (0.92)		15 (0.87)	2 (1.46)
α-glucosidase inhibitor	763 (5.19)		735 (5.12)			28 (8.05)		64 (3.45)		51 (2.97)	13 (9.49)
Insulin	5186 (35.29)		5029 (35.06)			157 (45.11)		N/A		N/A	N/A
GLP-1^o^ agonist	19 (0.13)		19 (0.13)			N/A		N/A		N/A	N/A
SGLT2^p^ inhibitor	340 (2.31)		331 (2.31)			9 (2.59)		N/A		N/A	N/A
Hypertension^q^											
Angiotensin II receptor blockers	6501 (44.24)		6328 (44.11)			173 (49.71)		116 (6.25)		105 (6.11)	11 (8.03)
ACE^r^ inhibitor	1314 (8.94)		1273 (8.87)			41 (11.78)		69 (3.72)		61 (3.55)	8 (5.84)
Calcium-channel blocker	6507 (44.28)		6343 (44.21)			164 (47.13)		282 (15.19)		255 (14.83)	27 (19.71)
Diuretics	4499 (30.62)		4392 (30.61)			107 (30.75)		104 (5.60)		94 (5.47)	10 (7.30)
Beta-blocker	4007 (27.27)		3925 (27.36)			82 (23.56)		1 (0.05)		1 (0.06)	N/A
Dyslipidemia^s^											
Statin	8248 (56.13)		8043 (56.06)			205 (58.91)		453 (24.41)		407 (23.68)	46 (33.58)
Fibrate	653 (4.44)		640 (4.46)			13 (3.74)		31 (1.67)		22 (1.28)	9 (6.57)
Ezetimibe	804 (5.47)		794 (5.53)			10 (2.87)		41 (2.21)		37 (2.15)	4 (2.92)
Antiplatelet^t^											
Aspirin	6218 (42.32)		6048 (42.16)			170 (48.85)		358 (19.29)		324 (18.85)	34 (24.82)
Clopidogrel	4174 (28.41)		4078 (28.43)			96 (27.59)		156 (8.41)		133 (7.74)	23 (16.79)
Cilostazol	1898 (12.92)		1813 (12.64)			85 (24.43)		74 (3.99)		60 (3.49)	14 (10.22)
Glycoprotein IIb/IIIa antagonist	197 (1.34)		193 (1.35)			4 (1.15)		N/A		N/A	N/A

a Group of patients with newly developed neurodegenerative disease within 3 years.

b HbA_1c_: glycated hemoglobin.

c HDL: high-density lipoprotein.

d LDL: low-density lipoprotein.

e AST: aspartate transaminase.

f ALT: alanine transaminase.

g GGT: gamma-glutamyl transferase.

h ALP: alkaline phosphatase.

i Ischemic heart disease, myocardial infarction, heart failure, atrial fibrillation, stroke, and cerebrovascular disease.

j N/A: not available.

k Peripheral vascular disease and amputation.

l ESRD: end-stage renal disease.

m Metformin, sulfonylurea, DPP-4 inhibitor, meglitinide, thiazolidinedione, α-glucosidase inhibitor, insulin, GLP-1 receptor agonist, and SGLT2 inhibitor.

n DPP-4: dipeptidyl peptidase-4.

o GLP-1: glucagon-like peptide-1.

p SGLT2: sodium-glucose cotransporter 2.

q Angiotensin II receptor blockers, ACE inhibitors, calcium channel blockers, diuretics, and beta-blockers.

r ACE: angiotensin-converting enzyme.

s Statin, fibrate, ezetimibe, and omega-3.

t Aspirin, clopidogrel, cilostazol, and glycoprotein IIb/IIIa antagonist.

### Comparisons of Prediction Model Performance

**Figure 2.**
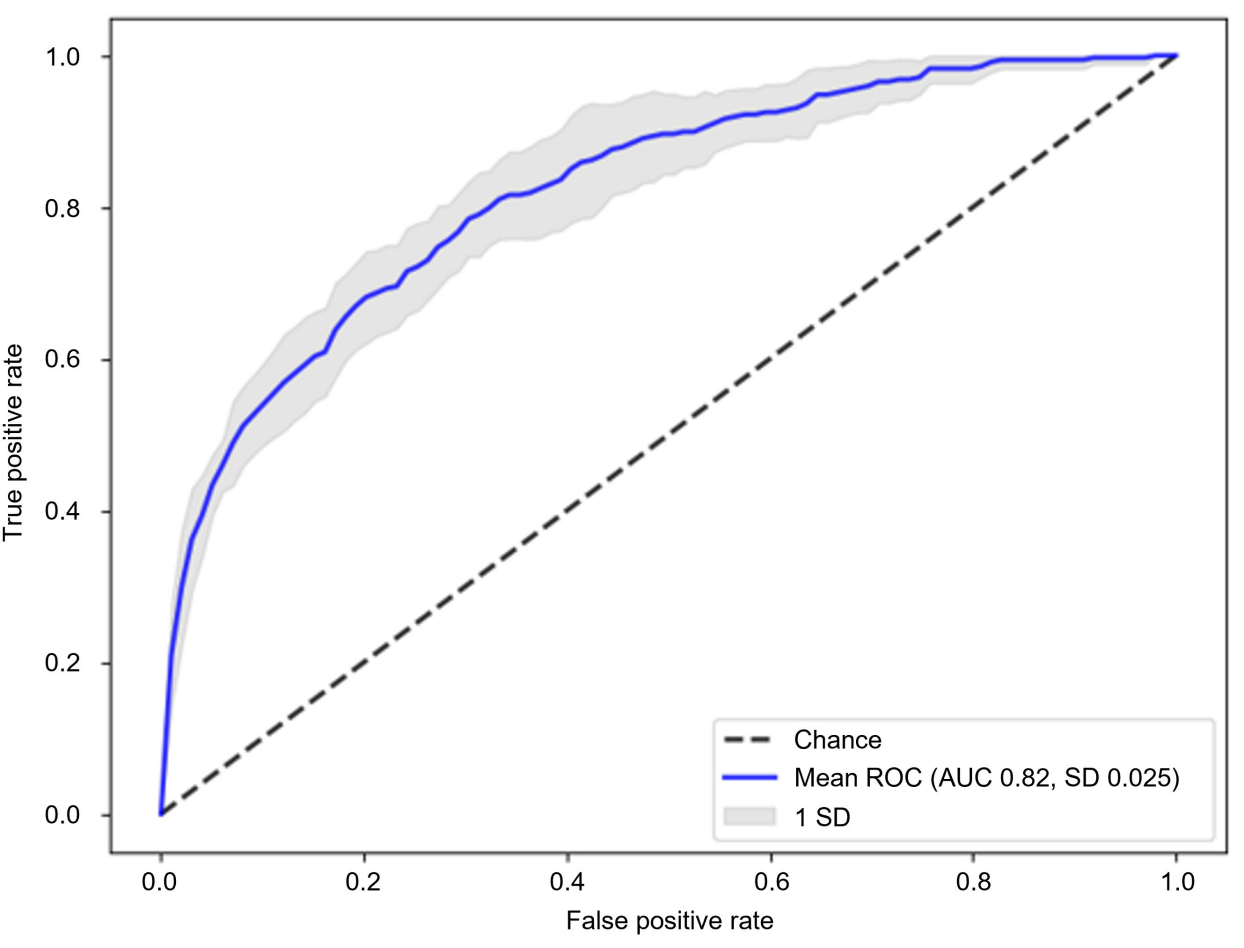
ROC curve of the XGBoost model. Mean ROC curve from 10-fold cross-validation on the original dataset. AUC: area under the ROC curve; ROC: receiver operating characteristic; XGBoost: extreme gradient boosting.

The XGBoost model performed well on the discovery set (AUROC 82.36 [95% CI 80.48‐84.25]; accuracy 75.13% [95% CI 74.10‐76.17]; sensitivity 71.00% [95% CI 66.83‐75.17]; specificity 75.23% [95% CI 74.16‐76.31]; balanced accuracy 73.12% [95% CI 71.06‐75.17]; precision 6.51% [95% CI 6.09‐6.94]; F1-score 11.93% [95% CI 11.18‐12.68]; and AUPRC 22.08 [95% CI18.04‐26.13]) (Table 2). When these models were applied to an external validation set, the XGBoost model achieved an AUROC of 71.67 (Table 2). Consequently, with consistent results in both independent datasets, the XGBoost model emerged as the best predictor of DR development within 3 years among patients with T2DM (Figure 2).

**Table 2.** Performance metrics of 5 different machine learning algorithms on the original and validation NIA^a^ datasets.

Model		AUROC^b^	Accuracy	Sensitivity	Specificity	Balanced accuracy	Precision	*F*_1_-score	AUPRC^c^
Original dataset, mean (95% CI)									
	XGBoost^d^	82.36 (80.48‐84.25)^e^	75.13 (74.10-76.17)^e^	71.00 (66.83-75.17)^e^	75.23 (74.16-76.31)^e^	73.12 (71.06-75.17)^e^	6.51 (6.09-6.94)^e^	11.93 (11.18-12.68)^e^	22.08 (18.04-26.13)^e^
CatBoost	82.12 (80.24-84.00)	73.51 (70.31-76.72)	73.01 (70.03-75.99)	73.53 (70.32-76.73)	73.27 (70.18-76.36)	6.45 (5.50-7.40)	11.84 (10.19-13.48)	21.70 (18.80-24.60)
LightGBM^f^	82.25 (79.83-84.67)	77.73 (76.95-78.50)	69.55 (65.03-74.06)	77.92 (77.09-78.76)	73.74 (71.63-75.84)	7.10 (6.69-7.52)	12.88 (12.14-13.63)	22.09 (18.85-25.33)
Random forest	80.84 (79.03-82.65)	73.55 (70.97-76.12)	73.01 (70.95-75.07)	73.56 (70.97-76.15)	73.28 (70.98-75.59)	6.39 (5.66-7.12)	11.74 (10.48-13.00)	18.91 (15.45-22.37)
AdaBoost	79.99 (76.32-83.67)	73.15 (69.06-77.23)	72.68 (69.20-76.17)	73.16 (69.05-77.26)	72.92 (69.21-76.63)	6.44 (5.33-7.54)	11.80 (9.88-13.72)	20.74 (16.62-24.87)
Validation dataset, mean									
	XGBoost	71.67^e^	65.14^e^	64.96^e^	65.15^e^	65.06^e^	12.94^e^	21.58^e^	16.57^e^
CatBoost	71.43	65.95	65.69	65.97	65.83	13.33	22.17	21.49
LightGBM	70.87	64.98	64.96	64.98	64.97	12.88	21.50	17.84
Random forest	68.12	62.39	62.04	62.42	62.23	11.63	19.59	15.89
AdaBoost	65.35	61.42	61.31	61.43	61.37	11.24	19.00	14.70

a NIA: National Information Society Agency.

b AUROC: area under the receiver operating characteristic.

c AUPRC: area under the precision-recall curve.

d XGBoost: extreme gradient boosting.

e Best-performing model.

f LightGBM: light gradient-boosting machine.

### Feature Importance

**Figure 3.**
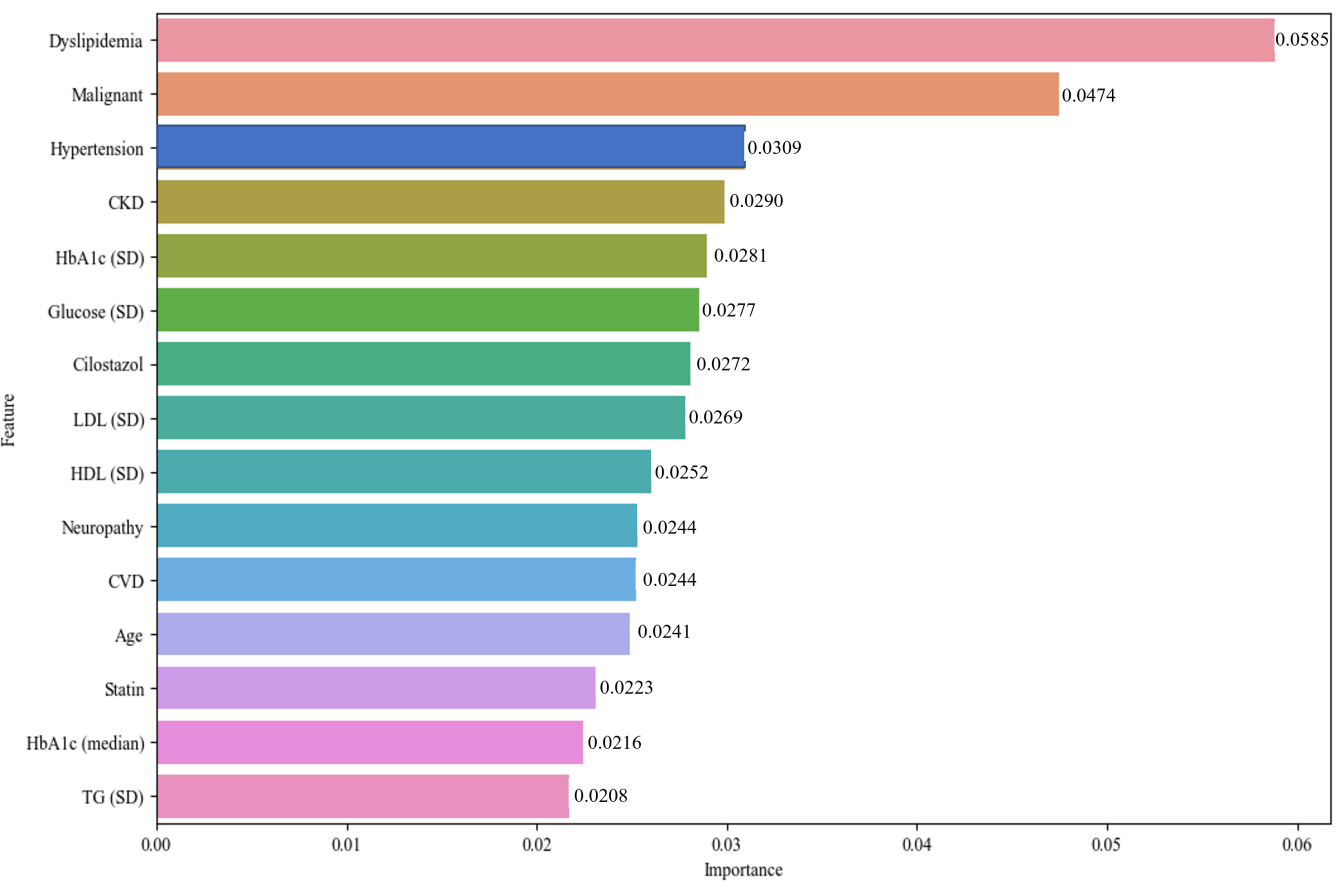
Top 15 important features in the XGBoost model. CVD: cardiovascular disease; CKD: chronic kidney disease; DDP: Dipeptidyl Peptidase; HbA_1c_: glycated hemoglobin; HDL: high-density lopoprotein; LDL: low-density lipoprotein; TG: triglyceride; XGBoost: extreme gradient boosting.

The XGBoost model identified dyslipidemia (0.0585) as the most significant factor in predicting the development of DR within 3 years in patients with T2DM (Figure 3). Following dyslipidemia, the most critical factors included malignancy (0.0474), hypertension (0.0309), CKD (0.0290), HbA_1c_ variability (SD) (0.0281), glucose variability (SD) (0.0277), cilostazol use (0.0272), low-density lipoprotein variability (SD) (0.0269), high-density lipoprotein variability (SD) (0.0252), neuropathy (0.0244), CVD (0.0244), age (0.0241), statin use (0.0223), median HbA_1c_ (0.0216), and triglyceride variability (SD) (0.0208). These factors were ranked in descending order based on their importance in predicting DR.

## Discussion

### Principal Findings

This study used data from 3 university hospitals in South Korea to conduct a simple and accurate assessment of ML-based risk prediction for DR development that can be universally applied to adults with T2DM. The AdaBoost, LightGBM, Random Forest, and XGBoost ensemble models performed well, with AUROC values of 82.36% (95% CI 80.48‐84.25). Dyslipidemia and cancer were the most important factors influencing the predictive model.

Several studies have investigated vision-threatening DR using deep learning (DL) approaches. Gulshan et al [15] used 9963 fundus images, achieving an AUROC of 0.999. Similarly, Ting et al [16] validated a DL system using 71,896 images, reporting an AUROC of 0.958 (95% CI 0.956‐0.961). Li et al [17] also validated a DL system with 35,201 images, obtaining an AUROC of 0.955. These studies reported a more robust performance of DL systems than human testing in various patient populations [15,18]. However, it is important to note that these investigations primarily focused on patients with severe vision-threatening DR, diagnosed through specialized ophthalmological care. Furthermore, a direct end-to-end training approach links the fundus image to the DR grade labels and may overlook critical lesion features due to the inherent black-box nature of DR classification models [19]. This limitation underscores the need for more comprehensive methodologies that can effectively capture and encode relevant lesion characteristics in the context of DR diagnosis.

This study offers the advantage of identifying the strong predictive performance of previously unseen data using a decision tree–based ensemble model that optimizes the combination of hyperparameters. Specifically, the findings suggest that DR can be reliably predicted in patients with T2DM using simple medical information obtained in primary care settings before ophthalmologist consultation and at the onset of visual symptoms. This approach can significantly reduce health care costs by enabling early detection and intervention. Gayathri et al [20] introduced a DR classification system based on various MLs, including multipath convolutional neural networks, random forests, and support vector machines. Similarly, Math et al [21] developed a DR classification system based on adaptive ML and obtained an AUC of 0.963.

This study proposed a new selection method for predicting DR with high accuracy and minimal predictors. The final dataset comprised 15 independent variables, and to identify the most significant risk factors of DR, ensemble models including the AdaBoost, LightGBM, Random Forest, and XGBoost were used to reduce the number of features among the numerous risk factors. The findings indicate that DR exhibited the strongest correlation with dyslipidemia, followed by cancer, hypertension, CKD, neuropathy, and CVD. These results are consistent with previously reported correlations between dyslipidemia [22,23], cancer [24], hypertension [25,26], CKD [27], neuropathy [28], and CVD [29]. This study underscores the importance of these correlations in understanding the multifactorial nature of DR. It highlights the necessity for comprehensive risk assessments in patients with DM to effectively mitigate the risk of developing DR. In addition, a higher BMI is commonly associated with an increased risk of complications in patients with DM, and studies have shown that DR can develop even in individuals with a BMI of approximately 25, as observed in the study cohort. This suggests that a lower BMI does not necessarily protect against DR and highlights the need for comprehensive risk assessment in all patients with DM, regardless of BMI.

The findings demonstrated that DR in patients with diabetes can be predicted using simple medical information in the primary care setting before the onset of visual impairment and before ophthalmological treatment, thus potentially dramatically reducing medical costs. Furthermore, this study highlights the generalizability of ML systems and their feasibility in scaling up screening programs while maintaining the standards of care. ML systems offer reasonably accurate predictions that can significantly improve settings where additional testing may take weeks or months.

Artificial intelligence (AI)–based technologies support testing for DR, which is an unmet public health concern [30,31]. The results demonstrated that AI-based personalized testing intervals can be incorporated to improve the efficiency, equity, and accessibility of DR testing [32]. In particular, the findings of this study could improve patient outcomes by enabling timely intervention, improving the understanding of contributing variables, and reducing the burden of DR complications in patients with T2DM. The use of AI in the new DR classification is promising and there is optimism regarding future guideline revisions for the use of AI in the management of DR [33].

### Limitations

First, as a cohort study conducted in hospitals in urban areas of a single ethnic group, the findings may not represent the entire population. Second, the diagnosis of DR relied on disease codes, which may lead to underestimation. Third, obtaining accurate information was difficult as a retrospective study based on hospital medical records. As no systematic referral tracking exists for the review of electronic medical records, a customized tracking process was implemented within the study. Fourth, the performance of this prediction model cannot be directly compared with existing prediction models. Additionally, the severity of DR was not reflected in the results. Fifth, this study could not confirm a causal relationship between the predictors used in the model and the occurrence of DR. Additional studies are needed to accurately identify the pathophysiological pathways and demonstrate the mechanisms of interaction between variables associated with DR and their impact on DR development. Finally, because the model operates over a wide range of retrospective scenarios, it may not work well if an unexpected situation occurs, such as the outbreak of a new infectious disease such as COVID-19, or if medical records are inconsistent or insufficient. Future studies should use additional models to reduce the dependence of supervised feature selection techniques on a wide range of training data.

This study is the first to apply an ML-based DR prediction system to a nationwide population with diabetes. The results indicate that the implementation of evidence-based, individualized preventive interventions can reduce the burden of DR in Korean patients with diabetes.

### Conclusions

This study used a hospital-based cohort to develop an ML-based prediction model to accurately predict the DR risk in a Korean population with T2DM. These results may improve patient outcomes by enabling timely interventions to prevent DR in patients with T2DM, enhancing the understanding of contributing variables, and reducing the burden of DR complications in patients with T2DM. The prediction model proposed in this study is expected to be competitive and cost-effective in preventing DR in patients with T2DM in South Korea and is expected to be widely used, especially in primary care settings.

## Abbreviations

AI: artificial intelligence
AUROC: area under the receiving operating characteristic
CKD: chronic kidney disease
CVD: cardiovascular disease
DL: deep learning
DM: diabetes mellitus
DR: diabetic retinopathy
HbA_1c_: glycated hemoglobin
*ICD-10*: *International Statistical Classification of Diseases, Tenth Revision*
LightGBM: light gradient-boosting machine
ML: machine learning
ROC: receiver operating characteristic
T1DM: type 1 diabetes mellitus
T2DM: type 2 diabetes mellitus
XGBoost: extreme gradient boosting
